# Supplementary figures and images for: A plant reovirus hijacks endoplasmic reticulum-associated degradation machinery to promote efficient viral transmission by its planthopper vector under high temperature conditions
Source: PLoS Pathog. 2021 Mar 1;17(3):e1009347. doi: 10.1371/journal.ppat.1009347 (PMC7951979; doi:10.1371/journal.ppat.1009347)

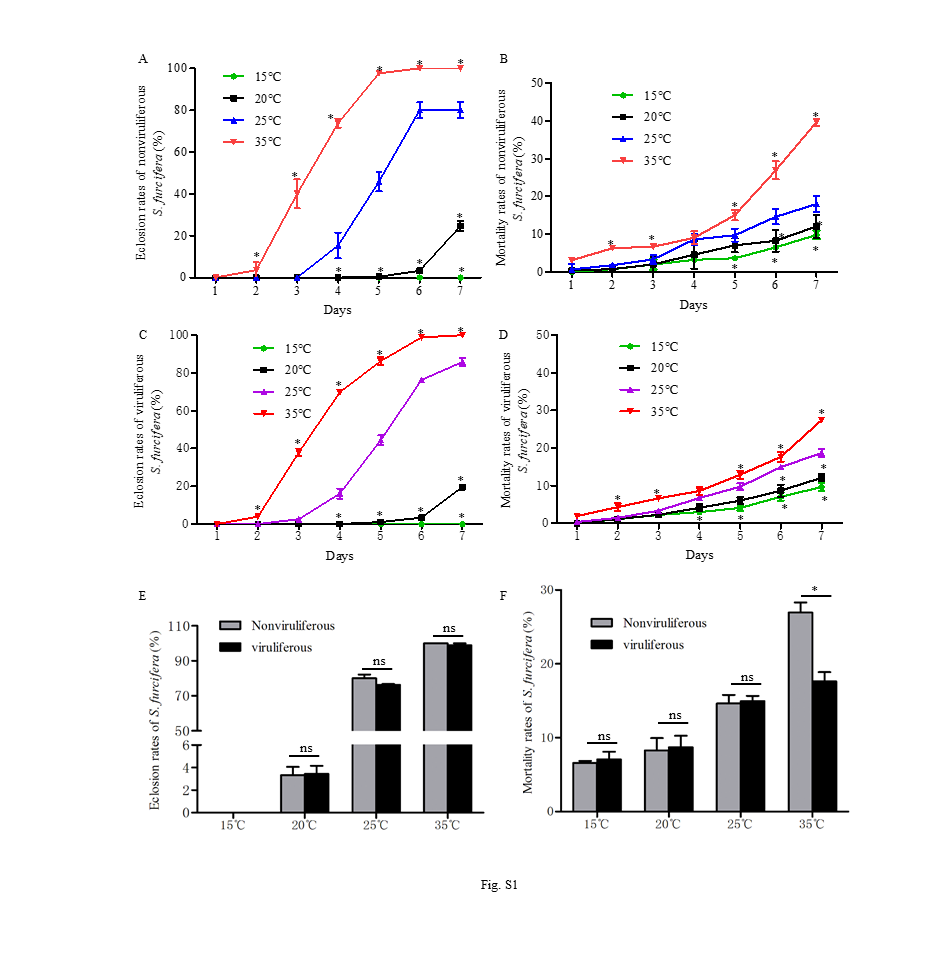

Supplement: S1 Fig — (A-D) The eclosion (A, C) and mortality (B, D) rates of nonviruliferous or viruliferous S. furcifera. Nonviruliferous or viruliferous fourth- to fifth-instar nymphs of S. furcifera were treated with temperatures at 15°C, 20°C, 25°C, or 35°C for different days, and the eclosion and mortality numbers were calculated daily. Each test contained three replicates and each replicate contained about 100 individuals of S. furcifera. Means (±SD) from three biological replicates are shown. The statistical significance in A-D are related to the 25°C control. *P<0.05. (E, F) The comparison of eclosion (E) or mortality (F) rates between nonviruliferous and viruliferous S. furcifera under different temperatures for 6 days. Each test contained three replicates and each replicate contained about 100 individuals of S. furcifera. Means (±SD) from three biological replicates are shown. *P < 0.05. ns, not statistically significant. (TIF) [file ppat.1009347.s001.TIF]

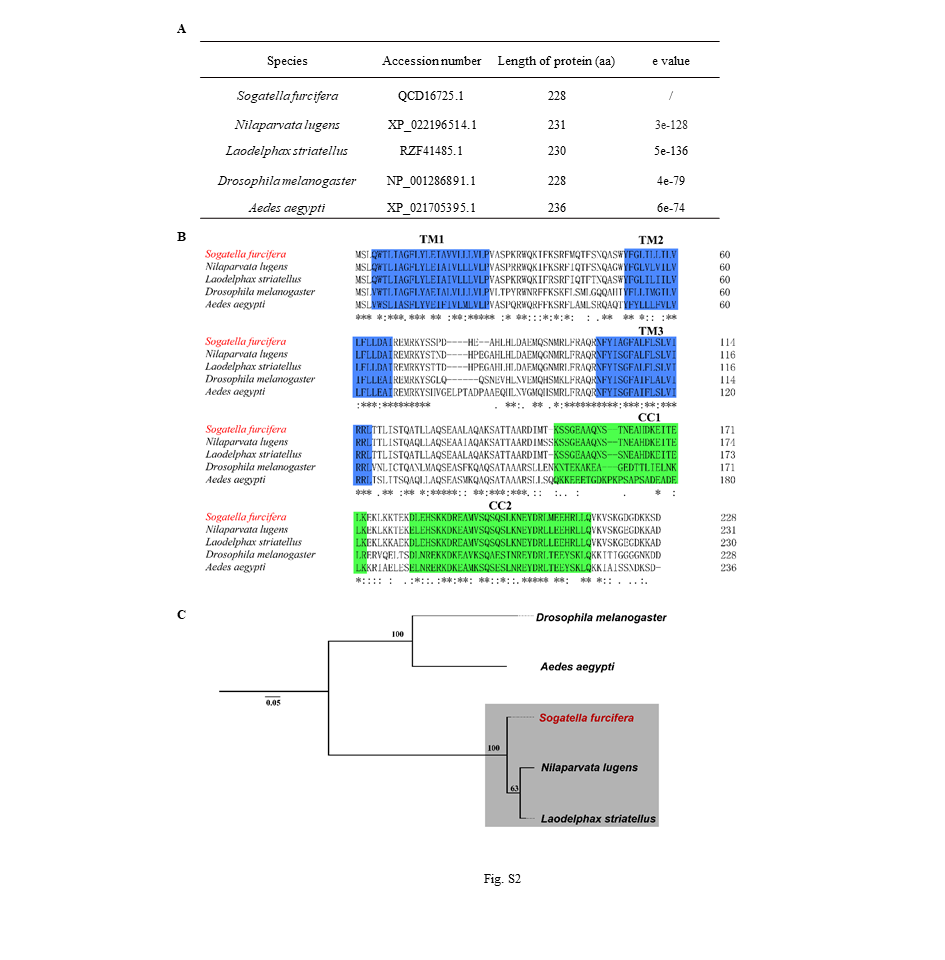

Supplement: S2 Fig — (A) The amino acid homology between BAP31 from S. furcifera and other insect species. (B) Comparison between deduced amino acid sequences of BAP31 from S. furcifera and other insect species. Three predicted transmembrane helices are highlighted in blue and labeled with TM1, TM2 and TM3 in the N-terminus. The two coiled coils are labeled with CC1 and CC2 and marked with green lines in the C-terminus. (C) Phylogenetic tree of BAP31 amino acid sequences from N. lugens, Laodelphax striatellus, Drosophila melanogaster and Aedes aegypti. Numbers at each branch indicate the percentage of times a node was supported in 1000 bootstrap replicates. (TIF) [file ppat.1009347.s002.TIF]

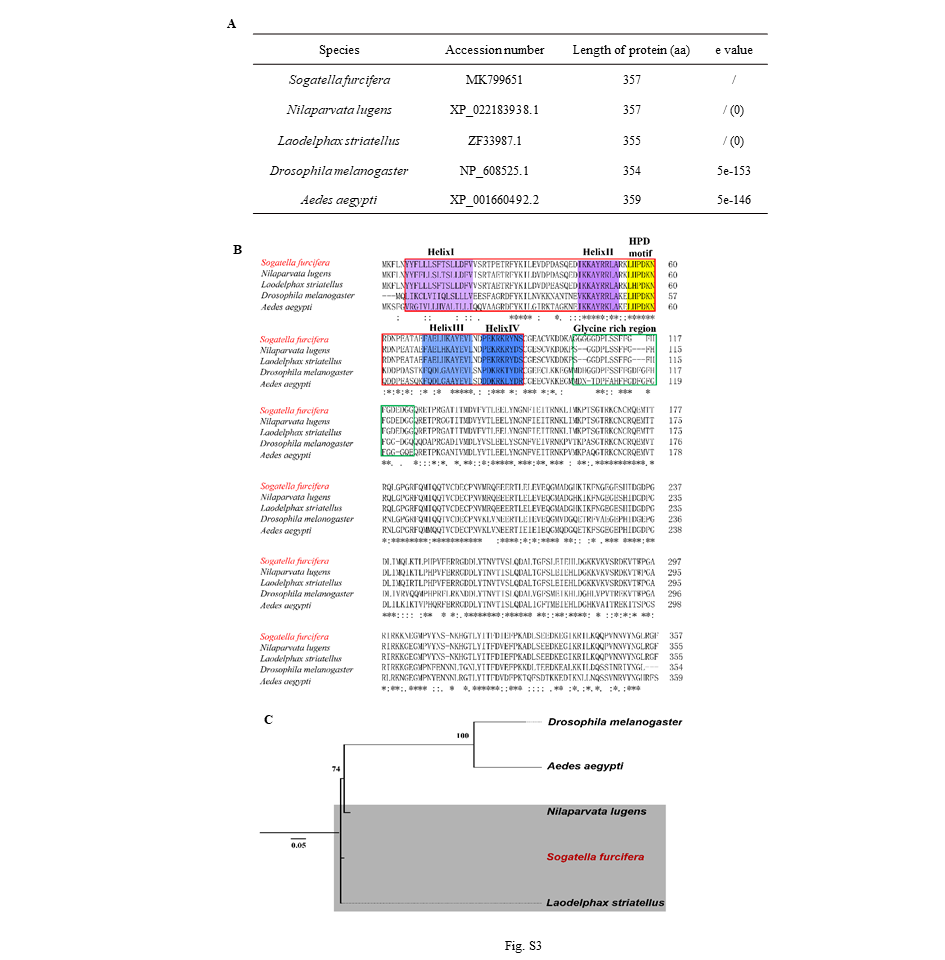

Supplement: S3 Fig — (A) The amino acid homology between DnaJB11 from S. furcifera and other insect species. (B) Comparison between deduced amino acid sequences of DnaJB11 from S. furcifera and other insect species. The conserved J-domain including Helix I region (highlighted with light purple), Helix II region (highlighted with purple), HPD motif (highlighted with yellow), Helix III region (highlighted with light blue), and Helix IV region (highlighted with blue) are marked by a red box, and the Glycine-rich regions are designated by a green box. (C) Phylogenetic tree of DnaJB11 amino acid sequences from S. furcifera, N. lugens, L. striatellus, D. melanogaster and A. aegypti. Numbers at each branch indicate the percentage of times a node was supported in 1000 bootstrap replicates. (TIF) [file ppat.1009347.s003.TIF]

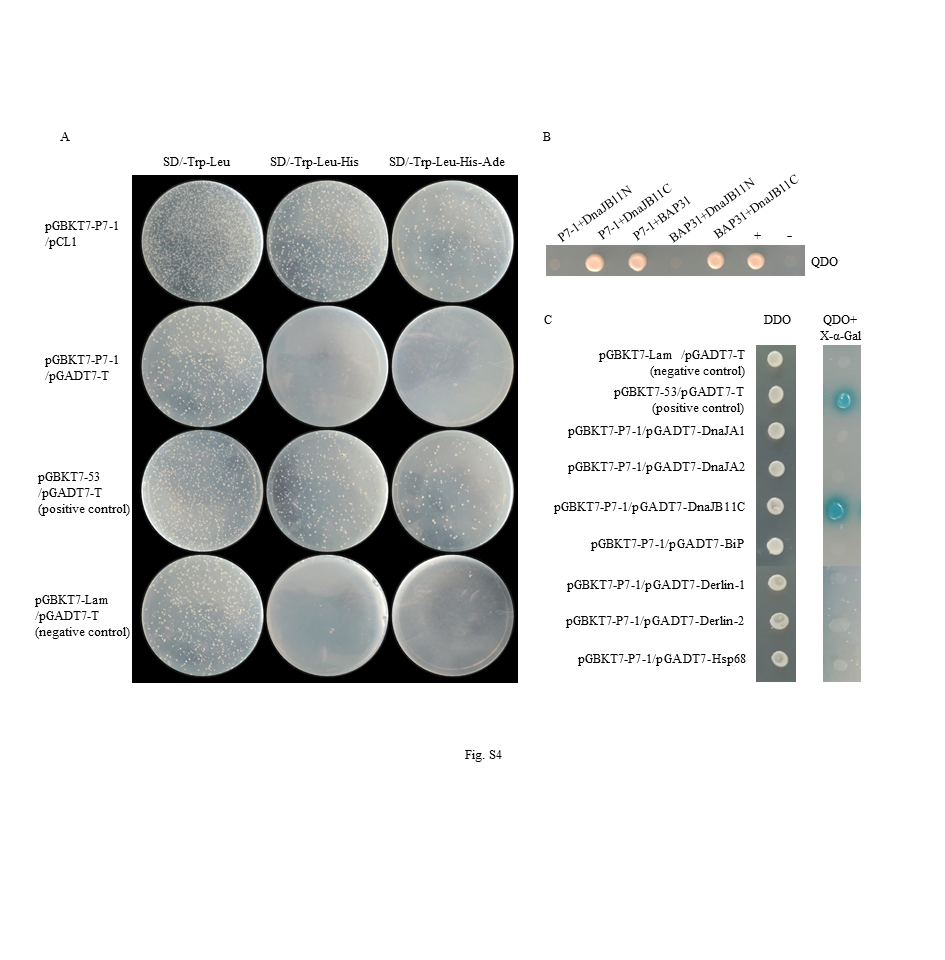

Supplement: S4 Fig — (A) The autoactivation test of P7-1 bait fusion plasmid. The pGBKT7-P7-1 was cotransformed with the control plasmid pCL1 or pGADT7-T and grown on selective SD medium. Coexpression of pGBKT7-P7-1 with pCL1 resulted in reporter gene activation as shown by growth of the yeast transformants, whereas co-expression of pGBKT7-P7-1 with pGADT7-T did not yield any yeast transformant growth on selective medium. pGBKT7-53 and pGADT7-T were used as positive controls; pGBKT7-Lam and pGADT7-T were used as negative controls. (B) The interactions of SRBSDV P7-1 with BAP31, DnaJB11N, or DnaJB11C of S. furcifera were tested by yeast two hybrid assay using a DUALmembrane starter kit. The transformants were plated on QDO culture medium. +, positive control (pBT3-STE/pOst1-NubI); −, negative control (pBT3-STE/pPPR3-N); P7-1+DnaJB11C, pBT3-STE-P7-1/pPR3-N-DnaJB11C; P7-1+DnaJB11N, pBT3-STE-P7-1/pPR3-N-DnaJB11N; P7-1+BAP31, pBT3-STE-P7-1/pPR3-N-BAP31; BAP31+DnaJB11N, pBT3-STE-BAP31/pPR3-N-DnaJB11N; BAP31+DnaJB11C, pBT3-STE-BAP31/pPR3-N-DnaJB11C. (C) The interactions of SRBSDV P7-1 with Derlin-1, Derlin-2, DnaJA1, DnaJA2, Hsp68, or BiP of S. furcifera were tested by yeast two-hybrid assay. Transformants were plated on DDO or QDO+X-α-Gal culture medium. (TIF) [file ppat.1009347.s004.TIF]

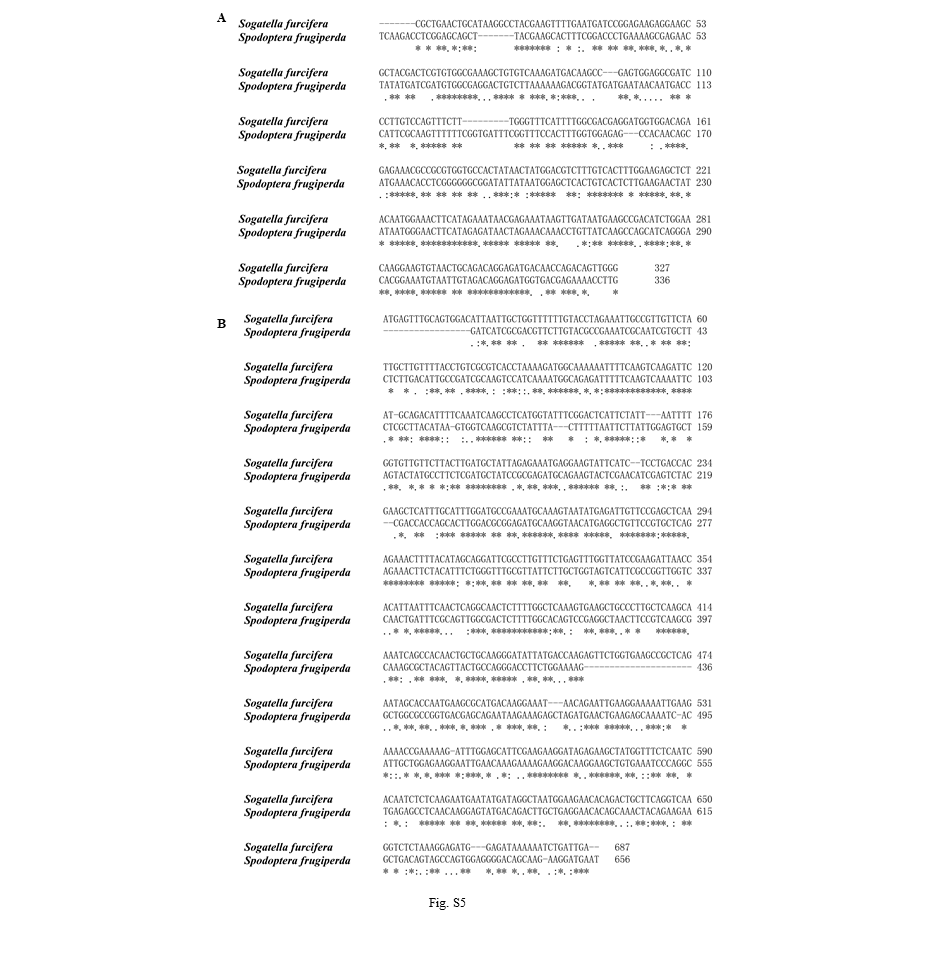

Supplement: S5 Fig — (A-B) Comparison between nucleotide sequences of DnaJB11 (A) and BAP31 (B) from S. furcifera and S. frugiperda. Nucleotide sequences of DnaJB11 and BAP31 from S. frugiperda shared 59% and 54% similarity with that from S. furcifera, respectively. (TIF) [file ppat.1009347.s005.TIF]
