# Supplementary material for: A plant reovirus hijacks endoplasmic reticulum-associated degradation machinery to promote efficient viral transmission by its planthopper vector under high temperature conditions
Source: PLoS Pathog. 2021 Mar 1;17(3):e1009347. doi: 10.1371/journal.ppat.1009347 (PMC7951979; doi:10.1371/journal.ppat.1009347)
Supplement: S1 Table — (DOC) [file ppat.1009347.s006.doc]

S1 Table Occurrence of P7-1 of SRBSDV in various tissues of insect vectors at 6 days padp, as detected by immunofluorescence microscopy.

| Temperatures  (˚C) | No. of positive insects with P7-1 in different tissues (n=30) | | | | | | | | | | |
| --- | --- | --- | --- | --- | --- | --- | --- | --- | --- | --- | --- |
|  | Midgut epithelium | | |  | Midgut muscle | | |  | Salivary gland | | |
|  | Expt I | Expt II | Expt  III |  | Expt  I | Expt  II | Expt  III |  | Expt  I | Expt  II | Expt  III |
| 15 | 6 | 5 | 5 |  | 0 | 1 | 0 |  | 0 | 0 | 0 |
| 20 | 9 | 7 | 10 |  | 5 | 4 | 4 |  | 0 | 0 | 0 |
| 25 | 2 | 0 | 3 |  | 19 | 22 | 20 |  | 15 | 13 | 12 |
| 35 | 0 | 0 | 0 |  | 27 | 26 | 24 |  | 20 | 18 | 21 |
