# Supplementary material for: A plant reovirus hijacks endoplasmic reticulum-associated degradation machinery to promote efficient viral transmission by its planthopper vector under high temperature conditions
Source: PLoS Pathog. 2021 Mar 1;17(3):e1009347. doi: 10.1371/journal.ppat.1009347 (PMC7951979; doi:10.1371/journal.ppat.1009347)
Supplement: S2 Table — (DOC) [file ppat.1009347.s007.doc]

S2 Table Transmission rates of SRBSDV by individual *S. furcifera* treated with different temperatures.

| Temperatures  (˚C) | No. of viruliferous insects (n=30) | | |  | No. of insects that transmitted SRBSDV  (n=30) | | | |
| --- | --- | --- | --- | --- | --- | --- | --- | --- |
|  | Expt  I | Expt II | Expt III |  | Expt  I | Expt  II | Expt III | Transmission rates (%) |
| 15 | 6 | 7 | 6 |  | 0 | 0 | 0 | 0 |
| 20 | 10 | 12 | 9 |  | 4 | 4 | 3 | 12.2 |
| 25 | 23 | 18 | 21 |  | 18 | 15 | 17 | 55.6 |
| 35 | 28 | 25 | 27 |  | 28 | 25 | 27 | 88.9 |
