# Supplementary material for: A plant reovirus hijacks endoplasmic reticulum-associated degradation machinery to promote efficient viral transmission by its planthopper vector under high temperature conditions
Source: PLoS Pathog. 2021 Mar 1;17(3):e1009347. doi: 10.1371/journal.ppat.1009347 (PMC7951979; doi:10.1371/journal.ppat.1009347)
Supplement: S3 Table — (DOC) [file ppat.1009347.s008.doc]

S3 Table Putative proteins of *S. furcifera* interacted with SRBSDV P7-1 in yeast-two hybrid system.

| **Sequence number** | **Accession number** | **BLASTX result** | **Species** | **e-value** | **ORF** | **Length of protein (aa)** |
| --- | --- | --- | --- | --- | --- | --- |
| 1 | XP_022196514.1 | B-cell receptor-associated protein 31 | *Nilaparvata lugens* | 3e-128 | full-length | 228 |
| 2 | XP_022183938.1 | DnaJ homolog subfamily B member 11 | *Nilaparvata lugens* | 0 | partial | 173 |
| 3 | KC122898.1 | Myosin RLC2 | *Nilaparvata lugens* | 4e-54 | partial | 169 |
| 4 | XP_022194790.1 | Peptidyl-prolyl cis-trans isomerase-like | *Nilaparvata lugens* | 3e-136 | full-length | 212 |
| 5 | XP_022196401.1 | Ubiquitin-protein E3 ligase | *Nilaparvata lugens* | 5e-79 | partial | 196 |
| 6 | XP_022200975.1 | 6-phosphogluconate dehydrogenase, decarboxylating isoform 1 | *Strongylocentrotus purpuratus* | 0 | full-length | 483 |
| 7 | EGI61990.1 | Trafficking protein particle complex subunit 9 | *Acromyrmex echinatior* | 3e-50 | partial | 149 |
| 8 | ABD98763.1 | Vacuolar ATPase G subunit-like protein | *Graphocephala atropunctata* | 2e-36 | full-length | 119 |
| 9 | EZA48348.1 | Ubiquitin thioesterase otubain-like protein | *Ooceraea biroi* | 3e-70 | partial | 237 |
| 10 | XP_014481934.1 | Tubulin alpha-1 chain-like | *Dinoponera quadriceps* | 0 | partial | 342 |
| 11 | XP_001951336.2 | Collagen alpha-1(IV) chain-like | *Acyrthosiphon pisum* | 2e-32 | partial | 230 |
| 12 | YP_009384892.1 | ATP synthase CF0 subunit I | *Dimeria ornithopoda* | 2e-30 | partial | 107 |
| 13 | AEM43833.1 | Cytochrome oxidase subunit I | *Sogatella furcifera* | 3e-21 | partial | 80 |
| 14 | XP_022192773.1 | Succinyl-CoA ligase subunit alpha, mitochondrial | *Nilaparvata lugens* | 8e-150 | full-length | 327 |
| 15 | XP_022207900.1 | Derlin-1、Der1-like domain family member 1 | Nilaparvata lugens | 2e-128 | full-length | 253 |
| 16 | XP_022192375.1 | Eukaryotic translation initiation factor 4E binding protein | Nilaparvata lugens | 9e-78 | full-length | 119 |
| 17 | XP_022183924.1 | Protein disulfide-isomerase | *Nilaparvata lugens* | 0 | full-length | 491 |
| 18 | BAN21151.1 | Mitochondrial ribosomal protein S25 | *Riptortus pedestris* | 4e-82 | full-length | 167 |
| 19 | EZA58108.1 | Tetraspanin -5D | *Cephus cinctus* | 4e-59 | partial | 127 |
| 20 | YP_008081177.1 | NADH dehydrogenase subunit 1 (mitochondrion) | *Sogatella furcifera* | 1e-103 | partial | 145 |
| 21 | XP_022198820.1 | U6 snRNA-associated Sm-like protein LSm7 | *Nilaparvata lugens* | 2e-69 | full-length | 122 |
| 22 | XP_022184553.1 | Fructose-bisphosphate aldolase | *Nilaparvata lugens* | 2e-158 | partial | 256 |
| 23 | KDR16890.1 | FK506-binding protein 2 | *Zootermopsis nevadensis* | 1e-34 | partial | 69 |
| 24 | AGH29104.1 | Cytochrome c oxidase subunit III | *Sogatella furcifera* | 9e-126 | full-length | 260 |
| 25 | XP_022199995.1 | 40S ribosomal protein SA | *Nilaparvata lugens* | 8e-158 | partial | 247 |
| 26 | AHB59659.1 | Odorant-binding protein 7 | *Sogatella furcifera* | 1e-139 | full-length | 258 |
