# Supplementary material for: A plant reovirus hijacks endoplasmic reticulum-associated degradation machinery to promote efficient viral transmission by its planthopper vector under high temperature conditions
Source: PLoS Pathog. 2021 Mar 1;17(3):e1009347. doi: 10.1371/journal.ppat.1009347 (PMC7951979; doi:10.1371/journal.ppat.1009347)
Supplement: S4 Table — (DOC) [file ppat.1009347.s009.doc]

S4 Table Primers used in this study.

| Primer name | Construct | Primer sequences | | | Restriction  enzyme |
| --- | --- | --- | --- | --- | --- |
|  |  | Forward |  | Reverse |  |
| Y1 | pGBKT7- P7-1 | 5'-CG***GGATCC***GTATGGATAGACCTGCTCGAGAACA-3' |  | 5'-CG***CTGCAG***GAGATGATGGAGATTCAAAAACAGG -3' | *Bam*HI/*Pst*I |
| Y2 | pGADT7-BAP31 | 5'-CG***GAATTC***ATGAGTTTGCAGTGGACATT-3' |  | 5-'CG***GGATCC***CGTTAATCAGATTTTTTATCTCCAT -3' | *EcoR*I/*Bam*HI |
| Y3 | pGADT7-DnaJB11C | 5'-CG***GAATTC***ATGGATGGCGATCCTGGTGATCT-3' |  | 5'-CG***GGATCC***CGTTAAAAACCACGCAGTCCATTAT-3' | *Eco*RI/*Bam*HI |
| Y4 | pGADT7-DnaJB11N | 5'-CG***GAATTC***ATGAAATTCCTCAACTATTA-3' |  | 5'-CG***GGATCC***CGTTATATGTGAGACTCGCCCTCTC-3' | *Eco*RI/*Bam*HI |
| Y5 | pGBKT7- BAP31 | 5'-CG***GAATTC***GTATGAGTTTGCAGTGGACATT-3' |  | 5-'CG***GGATCC***CGTTAATCAGATTTTTTATCTCCAT -3' | *EcoR*I/*Bam*HI |
| Y6 | pFastBac1-P7-1 | 5'-CG***GGATCC***ATGGATAGACCTGCT CGAGAACA-3' |  | 5'-GG***ACTAGT***TCAGATGATGGAGATTCAAAAACAGG-3' | *Bam*HI/*Spe*I |
| Y7 | pFastBac1-BAP31 | 5'-CG***GGATCC***ATGAGTTTGCAGTGGACATTAATTG-3' |  | 5'-GG***ACTAGT***TCAATCAGATTTTTTATCTCCATCTCCTT-3' | *Bam*HI/*Spe*I |
| Y8 | pFastBac1-DnaJB11C | 5'-CG***GGATCC***ATGGATGGCGATCCTGGTGATCT-3' |  | 5'-GG***ACTAGT***TTAAAAACCACGCAGTCCATTATATACAT-3' | *Bam*HI/*Spe*I |
| Y9 | T7-GFP | 5'-ATTCTCTAGAAGCTTAATACGACTCACTATAGGGACGTAAACGGCCACAAGTTC -3' |  | 5'-ATTCTCTAGAAGCTTAATACGACTCACTATAGGGAAGTCGTGCTGCTTCATGTG- 3' |  |
| Y10 | T7-BAP31 | 5'-ATTCTCTAGAAGCTTAATACGACTCACTATAGGGTCAAGTCAAGATTCATGCAG- 3' |  | 5'-ATTCTCTAGAAGCTTAATACGACTCACTATAGGGGCGCTTCATTGGTGCTATTCT-3' |  |
| Y11 | T7-DnaJB11 | 5'-ATTCTCTAGAAGCTTAATACGACTCACTATAGGGAAAGTGACATGGCCTGGAGC -3' |  | 5'-ATTCTCTAGAAGCTTAATACGACTCACTATAGGGAAAACCACGCAGTCCATTAT- 3' |  |
| Y12 | T7-Sf-BAP31 | 5'-ATTCTCTAGAAGCTTAATACGACTCACTATAGGGGATCATCGCGACGTTCTTGT- 3' |  | 5'-ATTCTCTAGAAGCTTAATACGACTCACTATAGGGCATCCTTCTTGCTGTCCCCT-3' |  |
| Y13 | T7-SF-DnaJB11 | 5'-ATTCTCTAGAAGCTTAATACGACTCACTATAGGGTCAAGACCTCGGAGCAGCT- 3' |  | 5'-ATTCTCTAGAAGCTTAATACGACTCACTATAGGGCGAGCCAAGGTTCCTCGTCAC- 3' |  |
| Y14 | q-P7-1 | 5'-AATGCGTACTCCCGACCTTC-3' |  | 5'-CGTCCTTCCGAAATAAAAGCG- 3' |  |
| Y15 | q-P9-1 | 5'-ATGGCAGACCTAGAGCGTAGAA-3' |  | 5'-CCGTCGTCGAGTAGGGGA - 3' |  |
| Y16 | q-BAP31 | 5'-GCTTGTTTTACCTGTCGCGT-3' |  | 5-'TGAGCTTCGTGGTCAGGAGA -3' |  |
| Y17 | q-DNA J | 5'-GGCCTGGAGCTCGCATACG-3' |  | 5'-ATCCTCTTGATACCTTCCTTGTCTT- 3' |  |
| Y18 | q-Actin | 5'-GCCGTCTTTCTTGGGTATGG-3' |  | 5'-AGGGCGGTGATCTCCTTCTG- 3' |  |
| Y19 | q-Sf-BAP31 | 5'-TCTCTTGACATTGCCGATCG-3' |  | 5-'GAAGTTAGCCTCGGACTGTG-3' |  |
| Y20 | q-Sf-DnaJB11 | 5'-CAAGACCTCGGAGCAGCT- 3' |  | 5'-GCCAAGGTTCCTCGTCAC- 3' |  |
| Y21 | q-Sf-Actin | 5'-CAGTGAGAGCCGTTTTGAG-3' |  | 5'-AGGGCATCTTGTCAGAGGGC- 3' |  |
| Y22 | His-P7-1 | 5'- AAGGCCATGGCTGATATCGGATCCATGGATAGAC  CTGCTCGAGA -3' |  | 5'- GCCGCAAGCTTGTCGACGGAGCTCTCAAGATGATGGA  GATTCAA - 3' |  |
| Y23 | GST-DnaJB11C | 5'- CCCCTGGGATCCCCGGAATTCATGGATCCTGGTGA  TCTGATCAT -3' |  | 5'- GTCACGATGCGGCCGCTCGAGTCAAAAAACCACGCA  GTCCATTA - 3' |  |
| Y24 | MBP-BAP31 | 5'- GGATTTCACATATGTCCATGAGTTTGCAGTGGACA  TT -3' |  | 5'- ACCTGCAGGGAATTCGGATCCTCAATCAGATTTTTTAT  CTC - 3' |  |
| Y25 | pGBKT7-P7-1N | 5'-CG***GGATCC***GTATGGATAGACCTGCTCGAGAA  CA-3' |  | 5'-CG***CTGCAG***GTTAGCTTTTCAAATTATTTTTAAT -3' | *Bam*HI/*Pst*I |
| Y26 | pGBKT7-P7-1C | 5'-CG***GGATCC***GTATGG ATGGAAGATGTTACTTTAGC  TCAA-3' |  | 5'-CG***CTGCAG***GTATGGATAGACCTGCTCGAGAACA-3' | *Bam*HI/*Pst*I |
| Y27 | pGADT7-BiP | 5'-CG***GAATTC***GACGAAGAAAAAGGGACAGTTATC-3' |  | 5'-CG***GGATCC***CGTTAAAGTTCGTCCTTGAGATCATCT -3' | *Eco*RI/*Bam*HI |

The T7 promoter sequence were underlined and the restriction enzyme site sequence were marked in italics and black.
