# Supplementary material for: A plant reovirus hijacks endoplasmic reticulum-associated degradation machinery to promote efficient viral transmission by its planthopper vector under high temperature conditions
Source: PLoS Pathog. 2021 Mar 1;17(3):e1009347. doi: 10.1371/journal.ppat.1009347 (PMC7951979; doi:10.1371/journal.ppat.1009347)
Supplement: S1 Data — (DOC) [file ppat.1009347.s010.doc]

**>Heat shock protein 68 of *Sogatella furcifera,* complete ORF 1941 bp**

ATGCCAGTGAAAACACCAGCGATTGGAATCGATCTCGGAACAACGTACTCGTGTGTCGGAGTGTTTCAACAGGGAAAAGTTGAGATTCTAGCCAACGACCAAGGAAACAGGACGACACCCAGCTATGTTGCATTCACGGACTCGGAGCGGCTCATCGGCGATGCGGCCAAAAACCAGGTCGCAATGAATCCGAAAAACACCATCTTCGACGCCAAACGTCTCATCGGTCGCCGTTTCGACGATCCGAAAATCACACAGGACATGAAGCACTGGCCCTTCAAGGTCTACAGCGACTGCGGCAAGCCCAAGATCGAGGTGGACTTCAAGGGCGAGGCGAAGAAGTTTTCTCCTGAGGAAATCAGCGCAATGGTGCTCACGAAAATGAAGGAGACGGCGGAAACCTACCTGGGTGCACCGGTGAAGGATGCAGTCATCACCGTTCCAGCCTACTTCAATGACTCACAGAGGCAGGCGACCAAGGATGCTGGAGCTATTGCAGGATTAAATGTGCTTCGAATTATCAACGAGCCTACAGCTGCTGCCCTTGCCTATGGGTTGGACAAGAACTTGAAAGGAGAGCGCAACGTACTCATTTTCGACCTGGGAGGAGGTACATTCGATGTCTCAATCCTAACCATCGACGAGGGCTCTCTATTTGAGGTGAGATCTACAGCAGGAGACACTCATCTGGGAGGAGAGGATTTTGACAACCGGCTTGTGGATCATCTATGTGAAGAATTCAAGAGGAAATATCGTAAAGACATCAAGTCCAATCCACGAGCTTTGAGAAGATTGAGAACTGCAGCTGAGAGGGCAAAGAGAACACTCTCTTCCAGCACTGAGGCCAGCATTGAAATAGATGCACTGTGTGAAGGAGTCGACTTCTACACCAAAGTATCACGAGCCCGATTTGAAGAATTGTGCGCAGATCTTTTCCGTTCCACACTGCACCCAGTTGAGAAAGCTCTGAACGACGCCAAGATGGACAAATCATCAATCCATGATGTCGTGCTAGTTGGAGGATCCACTCGTATACCCAAGATACAATCACTATTGCAGAACTTCTTCAATGGAAAATCACTCAACTTTTCAATCAACCCAGACGAAGCTGTCGCTTATGGAGCAGCTGTGCAAGCAGCTATACTCAGTGGAGATCAGAGCTCACAAATCCAGGACGTTCTTCTAGTTGATGTGACACCTCTCTCACTGGGCATTGAAACTGCAGGGGGAGTGATGGCGAAAATAATTGAACGCAACAGCAGAATTCCATGCAAGCAGTCACAAACCTTCAGCACTTATGCAGATAACCAGCCAGCAGTCACAATCCAAGTATTTGAAGGTGAGAGGGCGATGACTAAGGACAACAATCTACTGGGAACGTTTGATTTGACTGGAATACCACCAGCACCTAGAGGAGTACCGAAAATTGAGGTCACGTTTGATTTGGATGCAAACGGTATTTTGAATGTGTCGGCTAAGGAGACTGGAACTGGAATCACGAAGAATATTGTGATCAAGAACGATAAGGGACGTTTGTCCAAAGCTGATATTGATAGGATGGTGAACGAAGCTGAACAGTTTAAGGAGGAGGACGAAAAACAGAAGCAGAGAGTGAGCGCAAGGAATCAGCTGGAAAACTACATCTTCAGTGTGAAACAAGCGCTGGATGAAGCAGGAGGGAAACTGAGTGACGGTGATAAGTCGGAGGTGAAACAGAGATGCGACGAGTGCTTGCAGTGGCTTGATAACAACACACTCGCCGATAAGGAGGAGTTCGAGCACAAGCTGAAGGAGCTGCAGCAGCTATGCTCACCAATCATGGCCAAGATCTACTCGAGTGGAGCACCTGGAACTGGTGCTCAACCCACTTCCTGTGGACAACAGTTCAGCCAGGGCCAGTCATTCCCACGAAATGGACCTACTGTCGAGGAGGTAGATTAA

**>Derlin-1 of *Sogatella furcifera,* complete ORF 759 bp**

ATGACTGATATAGGCGAGTGGTTGAAATCTTTACCAATTTTCACCAGATATTGGTTACAATTGACTGTGGGTTTAACTCTGATTGCCCGCTTTGGTTTTGTGAACTATGAACATTTAGTTTTGGCGTATGAACCATTCATAAATAAATTCCAGATATGGAGACCAGTGACTGCTTTGTTCTACTACCCTTTGTCACCAAGTACTGGATTCCATTTCTTGATAAACTGCTACTTCTTGTAC

AACTACTCTCTTCGTCTTGAAAGAGATGTTTTTGGGGGAAGACCTGCTGATTATTTGTTCATGCTTTTTTTCAACTGGATATGCTGTGTCATCTGTTGTCTGTTTGCCCAAATTCCGGTAATGATGGATCCAATGGTGATGAGCATACTGTACATCTGGTGTCAACTGAATCCTGAGGTCATAGTGAGTTTCTGGTTCGGATCACGATTCAAAGCTACATATCTACCATGGGTGCTCTTCGGATTCAATCTTGTTGTTTCTGGCGGAGGTATACTTGAACTTTTTGGCATCATTGTTGGGCACACGTACTTCTTCCTCATGTTCAAATACCCCCAAGAGATGGGAGGACCACAGCTTATCAAAACACCGCAATTTCTGTACGACTGGTTCCCCAATCAGAGAACGATCCATGGCTTTGGATCAGTACCGACGCCCCGTGCTCCCCAGGCTCAGCCAGCAGCTGCTGATCCCCGCAGACATGACTGGGGACGCGGCCACGTTCTGGGAGACCGCCAGTGA

**>Derlin-2 of *Sogatella furcifera,* complete ORF 762 bp**

ATGGCTTACCAAACATTCCGTCAAGAATATCTTCAAATGCCAGTTGTGACCAGGGCATACACAACTGCATGTGTGATCACAACCTTGGCTGTCCAATTGAACTTGGTTTCGCCGTTCCAACTATACTTCAACCCTATCTTGATATTGAAACGGTATCAGATATGGCGAGTGCTGACAACGTTTCTGTTCTTTGGCACAGTGGGCTTCAATTTTTTCTTCAACATAATATTCACGTACCGCTACTGCAGAATGCTGGAGGAGGGCGCTTTCCGCGGTCAGACGGCTGACTTTGTCATGATGTTCATATTCGGTGGATGCTGTATGGTGTTCATTGCATTTTTCATTCATCTATTGTTCCTGGGTCAGGCATTCACGATTATGCTTGTTTATGTGTGGTCAAGAAGAAACCCTTACACTAGAATGAACTTCTTCGGACTCATGAATTTTCAGGCTCCCTACCTGCCATGGGTTCTGTTGGGTTTCTCGGTGCTGCTGGGTAACCCCGTCTGGGTCGACCTGATGGGTATGGCTGTTGGTCATCTGTACTATTACATTGAAGACGTCTTGCCCCAGATTCTTGGCGGATTCAGAGTGCTCAATACTCCACAGTTCATGAAACTGCTGTTCGATCCGCACGTGGAAGAGCCCGACTACACGCATGTGGCTGACGAACGACCGGGCGGCTACAACTGGCGAGGCGGATTGGACGAAATCCAACAGCAACAAGATCAGGCGGCCAATCAGCAGCCGCCTCCACAGTAG

**>DnaJA1 of *Sogatella furcifera,* complete ORF 1230 bp**

TATTTAATTGGAGAAGGAATTATGGTGAAAGAAACTACGTTCTATGATATTCTGGGTGTGAAGCCCAATTGCACGGGTGATGAATTGAAGAAGGCCTACAGAAAATTAGCGTTGAAATACCATCCAGATAAGAATGCCAATGAAGGTGATCGCTTCAAGCAGATTTCACAAGCCTATGAGGTGTTGTCCAATCCGGACAAGCGCGCCATATACGACAAGGGAGGCGAGCAGGCGCTGAAGGAAGGCTCCGGTGGACCCGGCGGCTTCGCCTCCAACCCGATGGACATTTTCGACATGTTCTTCTCTCCGTACGGTGGACGCGGCGGCCGACAGCGGGAGAGGCGCGGCAAGGACGTGCTGCATCACTTGTCCGTCTCACTGGAGGACCTCTATAAGGGCGCCGTGCGCAAGCTTGCTCTCGAGAAGAAGGTTATCTGCGAGAAGTGTGAAGGTCGCGGTGGCAAGAAGGGTGCCGTCGAGCCTTGTCCCACGTGCAAAGGAAAGGGTATTCAGATCCAAATTCACCAGCTGGCGCCTGGCATGGTTCAGCAGACGCAGTGCATTTGCCGTGACTGCGAGGGCCAGAAAGAGCGAATTAATCCCAAGGACCGGTGCAAAACCTGTAATGGAATGAAAGTAGTCCGCGAGCGAAAAATTCTGGAAGTGCACGTTGACCAGGGCATGGTCGACGGGCAGAAGATAGTGTTCAATGGCGAGGGCGACCAGGAGCCTGGTCTGAAGCCGGGAGACATCATCATTGTGCTGGACGAGATGGAGCATTCGCGCTTCAAGCGGTCTGGCAACGACCTCATTCTGCGCGAGAAGATCGAGCTGGTCGAGGCGCTGTGTGGGTTCCAGAAAGTTATTCAGACGCTCGACGACAGGGATCTGCTCATCAGCTCTCTGCCCGGATCGGTGATGAAGCACGGTGAGGTGAAGACAGTGATGGGCGAGGGTATGCCACAGCACAAGAACCCCTTTGAGAAGGGCCGTCTCATCATCCAGTTCCTTGTCGAGTTCCCCACCTCTCTGCCGCCCGAAGTTATTCCCGCACTCGAGGAGTGTCTACCCAAGAGACCCGAACAGATGATTCCAGACAACGCAGAAGAGTGCATTTTGGAGGACATGGATCCAGAGGCGGAGTCACACCGTCGCGACTCGAGAAACGCGTACGACGAGGACGAAATGGGAAGCCAGCAGGGCAACCGTGTGCAGTGCGCCACTCACTAA

**>DnaJA2 of *Sogatella furcifera,* complete ORF 1272 bp**

TTCACGTTTGCAGCTTGCAGGTTTGGACTGTGCAGATTGCAGTTCTTCGTCTCGAAGATGGCTGAAACTAAATTATATGATGTATTAGGTGTTAGTAGAAGCGCATCAGAGGCAGAATTGAAGAAGGCATATCGTAATCTTGCTAAAGAATTCCATCCTGACAAAAATCCTTACGCAGGTGACAAATTTAAAGAAATTTCATTCGCCTATGAAGTGCTTTCTGATCCAGAAAAACGATCCATATATGACAGACATGGAATCAAAGGAATTCAAGAAGGCGTTCAAGATTTTGGAGGGGAGGGTCTATTATTTTCCCAAATATTTTCAGGCGGCTTATTTGGCATGCCCAGTCGTATGTTTAATCAAAAAGGCGAAAACACTTTCCATTCTGTGAAAGTGACACTTGAAGAATTATATAATGGAAACAAGACGTCAGTGCTGCAGCTTAGTAAAAAGGTCCTGTGCGTCACTTGTAGTGGGAAGGGCTCAAAGTCAGGTCAAACATATCCTTGCCGGTCCTGTAAAGGGTATGGGGTTAGAATGGTTTATCGACACCTTCATCCATCAATGGCGCAACAGGTTCAAACCCAGTGCACAGATTGTGTTGGTTTGGGCTATGTAATTCACGAAATGGACCTCTGTCCGGGCTGTCGCGGTAAAAAAGTACTGAATGAAACAAAAATATTGAATGTGAAAATTGACAAGGGCATGGCTGATGGTCAAAAAATTTACTTCAGAGGCGAAGGACATCAGCAGCCTGACATGATTCCGGGTGATGTAATTCTAATTTTAAAACTGAAGCCACATGATCGATTCCAACGATCCGACAATGATCTGTATATGACTCAGAAAATAACGTTCACCGATGCTCTTTGTGGCT

TCTCTCTGGTTGTGAAACATCTCGACGGCCGAGATTTGCTCATCAATCATCCAGCGGGTCAAATTATCAAACCAGGTGATATTAAAGGTATTCGAGGGGAGGGTATGCCCGTTTACAGGAATCCATTTGAAAAAGGCAACCTCTATATCAAATTTGACGTTGAGTTCCCAGCGAATCATTCAATCAGTGAAGAGCATTTGAAGATGTTGGAGAGTCTTCTACCAGCCCGGCCTGCTGCAGTGATACCTCCGGCTGAAATGCTGGAAGAAGTGGACCTGTACGACTACGACCCAAATGACAGGAGAAATCAGCCGGCTGACAATGACGACGACGATGAATGCACCTACGAGGAGATGCACGCCGGGTCCATCCCTTCCTGTGCCTACCAATAA

**>BiP of *Sogatella furcifera,* complete ORF 1968 bp**

ATGAAACTACAATTATTTGGCGTTGTATGGGTCATGTGCCTGATGTGGCACAGGGCTATTGCAGCTGACGAAGAAAAAGGGACAGTTATCGGAATTGATTTAGGAACCACCTATTCTTGTGTTGGAGTGTACAGGAATGGCAGAGTTGAAATTATTGCCAATGACCAGGGTAACAGAATCACACCATCCTATGTGGCTTTCACCTTTGAAGGCGAAAGGTTGATTGGTGATGCTGCCAAGAACCAGCTCACCACAAATCCTGAGAACACCATCTTCGATGCCAAGCGTCTGATTGGTAGAGAATGGTCCGATGCTACTGTGCAGAGCGACATCAAATTCTTCCCATTCAAGGTTTTAGAGAAGAATAGCAAGCCACACATTCAGGTCAAGACCAGTCAGGGAAACAAGGTATTTGCTCCTGAAGAGGTTTCCGCTATGGTGCTTGGAAAGATGAAGGAAACAGCTGAAGCTTACTTGGGAAAGAAGGTTACCCACGCTGTTGTGACGGTACCGGCCTACTTCAACGATGCTCAGCGGCAGGCGACCAAGGATGCGGGAACCATTGCCGGCCTCAATGTGATGCGTATCATCAATGAGCCGACCGCCGCCGCTATTGCCTACGGCCTGGACAAGAGGGAGG

GCGAGAAGAACGTGCTCGTATTCGATCTGGGTGGTGGTACTTTTGATGTATCGCTCCTCACCATTGATAATGGAGTTTTTGAGGTGGTTTCGACGAACGGTGACACTCATTTGGGAGGCGAGGACTTTGACCAGCGTGTGATGGACCACTTCATCAAGCTGTACAAGAAGAAGAAGGGCAAGGACATTAGGAAGGACAACCGCTCTGTGCAGAAGCTGAGGAGAGAAGTGGAGAAGGCCAAGAGAGCGCTGTCCTCCAGCCATCAAGTCAGGATCGAAATCGAATCCTTCTACGAGAGCGATGACTTCTCTGAAACTCTCACCAGAGCCAAATTCGAGGAATTGAACATGGACTTGTTCAGATCAACAATGAAGCCGGTGCAGAAGGTTATTGAGGACGCCGACATGAACAAGAAGGACGTAAACGAGATTGTGCTGGTGGGTGGCAGCACGCGTATTCCCAAGGTGCAGCAGCTGGTCAAGGAGTTCTTCAACGGCAAGGAGCCATCGCGAGGTATCAATCCTGACGAGGCTGTAGCCTACGGAGCCGCCGTACAGGCCGGAGTCCTATCGGGAGAGGAGAAGACTGACGCCATTGTTCTGCTCGACGTCAACCCACTTACCATGGGCATTGAGACTGTGGGAGGTGTCATGACCAAGCTCATTCCGCGCAACACTGTCATCCCCACCAAGAAATCGCAGATCTTCTCCACCGCCTCTGACAACCAGCACACTGTTACCATTCAGGTATACGAAGGAGAACGACCAATGACAAAGGACAACCATCTTCTGGGAAAATTCGATCTTACCGGCATCCCACCAGCACCAAGAGGCGTGCCTCAGATTGAAGTCACATTCGAAATCGATGCCAACGGTATTCTACAGGTATCGGCTGAGGACAAGGGCACCGGCAACCGGGAGAAGATTGTGATCACCAACGACCAGAACCGTCTGACGCCCGAGGACATCGAGCGCATGATCAAGGACGCCGAGGCATTCGCCGACGACGACAAGAAGCTGAAGGAGCGCGTCGAGGCACGCAACGAGCTCGAGTCATACGCCTACTCGCTCAAGAACCAGCTCGGAGACAAGGAGAAGCTTGGCGCCAAGGTGAGCGATTCAGACAAGACAAAGATGGAGGAGGCGATCGACGAGAAAATCAAATGGCTGGAGGAGCACCAGGACGTGGACGCCGAGGAGTACAGGAAGGAGAAGAAGGAGCTGGAGGACATTGTGCAGCCAATCATCGCCAAGCTGTACCAGGGTACCGGAGGCCCCCCCCCCCCCGCCGGTGGCGCTCCCGAAGATGATCTCAAGGACGAACTTTGA
