# Supplementary material for: A plant reovirus hijacks endoplasmic reticulum-associated degradation machinery to promote efficient viral transmission by its planthopper vector under high temperature conditions
Source: PLoS Pathog. 2021 Mar 1;17(3):e1009347. doi: 10.1371/journal.ppat.1009347 (PMC7951979; doi:10.1371/journal.ppat.1009347)
Supplement: Attachment — Submitted filename: point-to-pont.pdf [file ppat.1009347.s011.pdf]

Point by point to Comments

We would like to thank the editor and three expert reviewers for their insightful comments and suggestions on the manuscript, as these comments and suggestions led us to an improvement of the manuscript. Our revision reflects all suggestions and comments. Detailed responses to the editors and reviewers are given below.

Editor's comments:

The manuscript by Yu and colleagues has been reviewed by 3 invited reviewers and myself. All agree that the presentation is good and the interactions shown and supporting figures are very nice. All reviewers make positive comments on the manuscript but also recommendations on specific things to address. Reviewer 1 makes a general point that the arguments given here by the authors that temperature etc. are applicable to all vector transmitted viruses is too general, really it only applies to this system. Reviewer 2 raises very important points about heat shock proteins/chaperones in general, and their roles in many cellular events. In this present manuscript also the authors show a correlation between temperature, their interaction and expression data and increased virus transmission by S. furcifera, but they do not demonstrate causality or specificity of the two proteins studied here. They do not show that either protein is definitively associated with virus transmission by S. furcifera, which is stated in the title and is their argument for the significance of this manuscript. They do show RNAi knockdowns and protein interaction/accumulation, perhaps if they could demonstrate specific knockdown effects on transmission efficiency that would give more definitive and mechanistic information.

Response: Many thanks for your positive evaluation of our work and valuable suggestions. Reviewer 1 points out a valuable advice as "Further work will follow to determine if the mechanisms described are conserved among tubule forming plant viruses or if this work is specific to this system." In the Discussion section, we have used a paragraph to discuss that the tubule formation induced by different plant viruses (such as SRBSDV P7-1, RBSDV P7-1, RDV Pns10, RGDV Pns11, and TSWV NSm) may also be mediated by Hsps through the protein quality control mechanism in the ERAD machinery. Further work will follow to determine if the virus-induced tubule formation in a temperature-dependent condition is conserved among tubule-forming plant viruses or is specific to SRBSDV-*S. furcifera* system. The related contents appear in lines 375-384.

Reviewer 2 raises very important points about heat shock proteins (Hsps)/chaperones in general, and their roles in many cellular events. In the Introduction section, we have added more information to show that many viruses such as TMV, TSWV, TYLCV, TBSV, and influenza A virus, hepatitis C virus and dengue virus require Hsps for viral infection. However, the mechanisms by which plant viruses activate the Hsps and their co-chaperones through the protein quality control mechanism in the ERAD machinery of insect vectors remain elusive. Furthermore, the mechanisms how plant viruses activate the Hsps and their

co-chaperones through the protein quality control mechanism in the ERAD machinery of insect vectors to adapt the high temperature stress also remain elusive. The related contents appear in lines 106-117.

Thank for your raising the important point about the causality or specificity of the two proteins BAP31 and DnaJB11 in the process of tubule formation or high temperature stress. We understand this problem is very important. In the revised version, we have added several new experiments to show the relationship among P7-1, BAP31, DnaJB11 and ER (Figures 2F-H and 3). In the Discussion section, we carefully analyze the causality of the two proteins BAP31 and DnaJB11 in the process of tubule formation or high temperature stress: "SRBSDV P7-1 is initially retained in the ER, and then secreted into the cytosol to assemble the tubules. BAP31 could act as an ER retention "receptor" for P7-1 by forming a complex with P7-1. DnaJB11 competes with BAP31 for binding P7-1, and thus the overexpression of DnaJB11 leads to the delivery of P7-1 from BAP31-P7-1 complex in the ER to DnaJB11-P7-1 complex. This process finally regulates the export of P7-1 from the ER and ensures the proper assembly of tubules. We thus deduce that BAP31 as a sorting factor may just transiently retain the newly synthesized P7-1 in the ER for further folding and assembly, and then deliver it to DnaJB11-containing chaperoning complex that determine the fate of P7-1. SRBSDV infection inhibits BAP31 expression but upregulates DnaJB11 expression, while BAP31 negatively regulates the expression of DnaJB11 through their direct interaction. Thus, the down-regulation of BAP31 expression during viral infection in turn promotes DnaJB11 expression and tubule assembly, finally facilitating viral transmission by *S. furcifera*. Taken together, we show that a fine virus-mediated regulation of protein quality control has evolved for activation of DnaJB11 for ensuring the proper assembly of virus-induced tubules to support viral propagation and transmission by insect vectors. This newly identified protein quality control pathway via regulation of BAP31 and DnaJB11 expression would play important roles in ensuring the proper protein function of P7-1 of SRBSDV under high temperature stress in viruliferous *S. furcifera*. High temperature stress further inhibits BAP31 expression but upregulates DnaJB11 expression during SRBSDV infection of *S. furcifera*, finally facilitating the assembly of more P7-1 tubules, ultimately facilitating highly efficient viral transmission by insect vectors in high temperature." The related contents appear in the Discussion section (lines 330-373).

Thank for your raising the important point about the transmission ability of *S. furcifera* after the knockdown of BAP31 or DnaJB11. In the revised version, we have added this experimental result as "The knockdown of DnaJB11 expression significantly decreased, while the knockdown of BAP31 expression significantly increased viral transmission rates by *R. dorsalis* into rice plants." The related contents appear in Figure 6G and in lines 281-283.

Reviewer's comments:

Part I – Summary

Reviewer #1: This paper describes new interactions between the proteins of Southern Rice Black Streaked Dwarf Virus and its host, Sogatella furcifera. This is an advancement in our understanding of tubule formation in a temperature dependent conditions. Further work will follow to determine if the mechanisms described are conserved among tubule forming plant viruses or if this work is specific to this system. Due to previously described articles, it suggests that Tomato spotted wilt virus may also have some similar interactions with DNAJ proteins. The paper is executed well with some minor writing flaws and some further analysis that would be helpful. Some editing of the figures will help make it even more beautiful.

Response: Thanks for your valuable suggestion. The nonstructural proteins of rice reoviruses such as SRBSDV P7-1, RBSDV P7-1, RDV Pns10, and RGDV Pns11 have similar secondary structures and form the tubular structures, which facilitate viruses to overcome the tissue or membrane barriers in respective insect vectors. Furthermore, the movement protein NSm of TSWV, a plant bunyavirus, forms the similar virus-containing tubules and interacts with plant DnaJ family proteins. Thus, the tubule formation induced by different plant viruses may also be mediated by Hsps through the protein quality control mechanism in the ERAD machinery. Further work will follow to determine if the virus-induced tubule formation in a temperature dependent condition is conserved among tubule-forming plant viruses or is specific to SRBSDV-*S. furcifera* system. The related descriptions in the Discussion section appear in lines 375-384. Furthermore, we have carefully revised the full text and the figures of the MS.

Reviewer #2: The manuscript entitled with "A plant reovirus hijacks endoplasmic reticulum-associated degradation machinery to promote efficient viral transmission by its planthopper vectors under high temperature conditions" by Yu et al. reported that southern rice black-streaked dwarf virus (SRBSDV) could hijack endoplasmic reticulum-associated degradation (ERAD) machinery for ensuring the proper assembly of abundant virus-induced tubules to support efficient viral transmission by Sogatella furcifera. In the present study, the authors identified DnaJB11 and BAP31 by using SRBSDV P7-1 protein as bait in the yeast two hybrid screening. They showed that P7-1 protein interacts directly with C-terminal domain of DnaJB11 and recruits the protein into P7-1 induced tubules to support efficient viral transmission; in contrast, BAP31 plays a negative role in the tubule assembly via downregulating DnaJB11 expression. They found high temperature increased the transcription expression level of DnaJB11 and reduced the BAP31 transcription. The findings that viral protein interact with similar heat shock protein 40 DnaJ to facilitate virus infection have been reported in many previous studies. DnaJ has also been shown to interact with ToMV MP and facilitate ToMV cell-to-cell movement and systemic infection. The correlation between heat shock proteins with the increased temperature-related virus transduction of cells has also been demonstrated. Overall, the experiments were well performed and data are beautiful, but the results are predictable as DnaJB11 would function as a co-chaperone. The results clearly

showed that molecular co-chaperone DnaJB11 helps the formation of viral tubular structure. However, the mechanisms how SRBSDV infection upregulate DnaJB11 and downregulate BAP31 at high temperature are still not well answered. DnaJ/Hsp40 family contains large members that are involved in a wide range of cellular events, including protein folding and oligomeric protein complex assembly. The present study hasn't provided the clear evidence showing that DnaJB11 is a component of ERAD.

Response: Thanks for these valuable comments and important suggestions. About the roles of heat shock proteins (Hsps)/chaperones in general in many cellular events, we have revised the manuscript and added more information to show that many viruses such as TMV, TSWV, TYLCV, TBSV, and influenza A virus, hepatitis C virus and dengue virus require Hsps for viral infection. We also have added more information to show that viral infection would increase the high temperature tolerance of insect vectors or plant hosts by stimulating the expression of Hsps. However, the mechanisms by which plant viruses activate the Hsps and their co-chaperones through the protein quality control mechanism in the ERAD machinery of insect vectors remain elusive. Furthermore, the mechanisms how plant viruses activate the Hsps and their co-chaperones through the protein quality control mechanism in the ERAD machinery of insect vectors to adapt the high temperature stress also remain elusive. The related contents appear in lines 93-117 in the Introduction section.

About the mechanisms how SRBSDV infection upregulate DnaJB11 and downregulate BAP31 at high temperature, we understand this problem is very important. In the revised version, we have added several new experiments to show the relationship among P7-1, BAP31, DnaJB11 and ER (Figures 2F-H and 3). In the Discussion section, we carefully analyze the causality of the two proteins BAP31 and DnaJB11 in the process of tubule formation or high temperature stress: "SRBSDV P7-1 is initially retained in the ER, and then secreted into the cytosol to assemble the tubules. BAP31 could act as an ER retention "receptor" for P7-1 by forming a complex with P7-1. DnaJB11 competes with BAP31 for binding P7-1, and thus the overexpression of DnaJB11 leads to the delivery of P7-1 from BAP31-P7-1 complex in the ER to DnaJB11-P7-1 complex. This process finally regulates the export of P7-1 from the ER and ensures the proper assembly of tubules. We thus deduce that BAP31 as a sorting factor may just transiently retain the newly synthesized P7-1 in the ER for further folding and assembly, and then deliver it to DnaJB11-containing chaperoning complex that determine the fate of P7-1. SRBSDV infection inhibits BAP31 expression but upregulates DnaJB11 expression, while BAP31 negatively regulates the expression of DnaJB11 through their direct interaction. Thus, the down-regulation of BAP31 expression during viral infection in turn promotes DnaJB11 expression and tubule assembly, finally facilitating viral transmission by *S. furcifera*. Taken together, we show that a fine virus-mediated regulation of protein quality control has evolved for activation of DnaJB11 for ensuring the proper assembly of virus-induced tubules to support viral propagation and transmission by insect vectors. This newly identified protein quality control pathway via regulation of BAP31 and DnaJB11 expression would play important roles in ensuring the proper protein function of P7-1 of SRBSDV under high temperature stress in viruliferous *S. furcifera*. High temperature

stress further inhibits BAP31 expression but upregulates DnaJB11 expression during SRBSDV infection of *S. furcifera*, finally facilitating the assembly of more P7-1 tubules, ultimately facilitating highly efficient viral transmission by insect vectors in high temperature.” The related contents appear in the Discussion section (lines 330-373).

About the evidence showing that DnaJB11 is a component of ERAD, we have cited more references to show that DNAJB11 is a co-chaperone of the ER HSP70 BiP and is a component of ERAD. Furthermore, we have confirmed that DnaJB11 is associated with ER together with BAP31 through their direct interaction, which further proves that DnaJB11 is a component of ERAD. The related contents appear in Figures 3G, and in lines 101-104 in the Introduction section.

Reviewer #3: Yu et al. report in this manuscript that Southern rice black-streaked dwarf virus (SRBSDV), a plant reovirus, is able to hijack ER degradation machinery thus promoting viral transmission by the insect vector (a planthopper) under high temperature conditions. This phenomenon is mediated by the differential interactions between a heat shock protein (DnaJB11) and an ER membrane protein (BAP31) from the insect side and the SRBSDV tubule protein P7-1. The final result is that at high temperatures, the virus is able to ensure the proper assembly of tubules needed for viral transmission by the insect. The authors also showed that transmission rates were higher at high temperatures, linking these results to the more frequent presence of some plant virus epidemics in hot-temperature areas (Btw, they missed the opportunity of briefly discuss the possible influence of the climate change on this). I think the authors used appropriate methodologies to demonstrate their claims, including Y2H, co-immunoprecipitation, as well as molecular and cellular approaches. They also knockdown DnaJB11 and BAP31 expression by RNAi to study tubule formation. The manuscript is in general well written and figures are correct (although a number of minor changes to them are suggested below). In summary, I believe that this work was well executed and could be of interest to an important number of PLOS Pathogens readers.

Response: Thanks for the positive comments. In the Discussion section, we briefly discuss that global warming may drive vector-borne viral pathogens emergence, expansion and epidemic in the nature, which appears in lines 375-384.

Part II – Major Issues: Key Experiments Required for Acceptance

Reviewer #1: All experiments that I would like to see are present.

Response: Thanks for the positive comment.

Reviewer #2: Special concerns:

1. The exact mechanism how DnaJB11 promotes and BAP31 inhibits P7-1 tubule assembly is still not well answered. Particularly, how BAP31 downregulate the transcriptional level of DnaJB11? As the authors mentioned, DnaJB11 and Hsp70 maybe work collaboratively to

ensure that the membrane protein P7-1 can be properly folded to assemble tubules. The upregulation of DnaJB11 maybe just stabilize the P7-1 protein. This is supported by the finding that the P7-1 protein accumulation level was decreased in dsDnaJB11 treated S. furcifera compared with that treated with dsBAP31 as shown in Fig. 4E and 4F, I would suggest the authors investigating whether HSP70 really contribute this process by using DnaJ mutant in HPD motif tripeptide of histidine (His), proline (Pro) and aspartic acid (Asp) that are crucial to deliver substrates to HSP70 and bind HSP70 to stimulate its ATPase activity.

Response: Thanks for these valuable comments. How SRBSDV infection upregulates DnaJB11 and downregulates BAP31? Particularly, how BAP31 downregulates the transcriptional level of DnaJB11? We understand these problems are very important. In the revised version, we have added several new experiments to show the relationship among P7-1, BAP31, DnaJB11 and ER (Figures 2F-H and 3). In the Discussion section, we carefully analyze the causality of the two proteins BAP31 and DnaJB11 in the process of tubule formation: “SRBSDV P7-1 is initially retained in the ER, and then secreted into the cytosol to assemble the tubules. BAP31 could act as an ER retention “receptor” for P7-1 by forming a complex with P7-1. DnaJB11 competes with BAP31 for binding P7-1, and thus the overexpression of DnaJB11 leads to the delivery of P7-1 from BAP31-P7-1 complex in the ER to DnaJB11-P7-1 complex. This process finally regulates the export of P7-1 from the ER and ensures the proper assembly of tubules. We thus deduce that BAP31 as a sorting factor may just transiently retain the newly synthesized P7-1 in the ER for further folding and assembly, and then deliver it to DnaJB11-containing chaperoning complex that determine the fate of P7-1. SRBSDV infection inhibits BAP31 expression but upregulates DnaJB11 expression, while BAP31 negatively regulates the expression of DnaJB11 through their direct interaction. Thus, the down-regulation of BAP31 expression during viral infection in turn promotes DnaJB11 expression and tubule assembly, finally facilitating viral transmission by S. furcifera. Taken together, we show that a fine virus-mediated regulation of protein quality control has evolved for activation of DnaJB11 for ensuring the proper assembly of virus-induced tubules to support viral propagation and transmission by insect vectors. This newly identified protein quality control pathway via regulation of BAP31 and DnaJB11 expression would play important roles in ensuring the proper protein function of P7-1 of SRBSDV under high temperature stress in viruliferous *S. furcifera*.” The related contents appear in the Discussion section (lines 330-357).

Whether HSP70 really contributes the process of BAP31-DnaJB11-mediated P7-1 tubule formation? DnaJB11 generally binds to the membrane proteins and delivers them to ER Hsp70 chaperone BiP for ATP-dependent chaperoning; however, DnaJB11 also can binds directly to unfolded protein clients in the ER without delivering to Bip. Yeast two-hybrid assay shows that BiP of *S. furcifera* does not directly interact with SRBSDV P7-1, suggesting that Bip may not be involved in the biogenesis of P7-1 formation. The related contents appear in Figure S5 and in lines 347-357.

2. The authors proposed that P7-1/DnaJB11 interaction was stronger than either the P7-1/BAP31 or BAP31/DnaJB11 interactions, indicating that P7-1 could outcompete binding with DnaJB11 and alter DnaJB11 ER localization to form tubules, however, they came to the conclusion only from yeast two hybrid and the evidence is far from enough. I would suggest the authors using some other methods like competitive pull-down assay to support this hypothesis. DnaJB11 maybe just help P7-1 protein to be more stable and this will cause the yeast grow better.

Response: Thanks for the comment. In the revised version, the competitive interactions among P7-1 of SRBSDV, BAP31 and DnaJB11C were detected by pull-down assay. The related content appears in Figure 2F and 2G.

3. Figure 6D and 6E, the authors performed interactions assay among SRBSDV P7-1, DnaJB11 and BAP31 in Sf9 cells. Indeed, they used DnaJB11C to replace wt- DnaJB11. I suggest adding a wt-DnaJB11 control to be consistent with the electron microscopy immunogold labeling shown in Figure 6F.

Response: We agree with this comment and followed. The wt-DnaJB11 was added in Figure 3I.

4. Rice black-streaked dwarf virus (RBSDV) is transmitted by Laodelphax striatellus. L. striatellus is adapted to the temperature below 28 degree. We would like to know whether the DnaJB11 is temperature-dependent in transmission of RBSDV by L. striatellus.

Response: Thanks for the comment. We have used yeast two-hybrid system to confirm that RBSDV P7-1 also can interact with DnaJB11 of *L. striatellus*. Owing to Covid-19 and other season conditions, we still do not have new RBSDV-infected rice plants and thus cannot try the experiments about temperature-dependent in transmission of RBSDV by *L. striatellus*. In Discussion section, we have analyzed the possible conserved mechanism for virus-induced tubule formation by activation of Hsp. The nonstructural proteins of rice reoviruses such as SRBSDV P7-1, RBSDV P7-1, RDV Pns10, and RGDV Pns11 have similar secondary structures and form the tubular structures, which facilitate viruses to overcome the tissue or membrane barriers in respective insect vectors. Furthermore, the movement protein NSm of TSWV, a plant bunyavirus, forms the similar virus-containing tubules and interacts with plant DnaJ family proteins. Thus, the tubule formation induced by different plant viruses may also be mediated by Hsps through the protein quality control mechanism in the ERAD machinery. Further work will follow to determine if the virus-induced tubule formation in a temperature dependent condition is conserved among tubule-forming plant viruses or is specific to SRBSDV-*S. furcifera* system. The related descriptions in Discussion section appear in lines 375-384.

Reviewer #3: I have only a suggestion for a new experiment to be included in the manuscript. The authors analyzed eclosion, mortality rates and development of the insect at different

temperatures. Also, they studied the virus infection rates in the insect at different temperatures. However, eclosion/mortality/ development could be analyzed also in viruliferous insects to give a more complete picture of the virus/insect vector/temperature interactions. In the case this information has been published elsewhere, it should be mentioned.

Response: Thanks for the comment. We have provided the eclosion and mortality rates of viruliferous insects in S1 Fig.

Part III – Minor Issues: Editorial and Data Presentation Modifications

Reviewer #1: Suggest title remove the S from the planthopper vector to indicate a single species is being studied.

Response: We have revised this.

• Line 141—This would be better to say “ ···we find that a fine-regulation has evolved for strong…” remove the been.

Response: We have revised this in the full text.

• Section: Interaction of SRBSDV P7-1 with ERAD…. Beginning on Line 166

o Of the 116 colonies sequenced- how many were repeats of each other, how many represent unique clones? Of those that are unique, were only 26 annotated? How many were you not able to annotate? Of those non-annotated, was it clear that there was a protein present and it is unique to S. furcifera?

Response: The total 116 positive colonies were sequenced. Of 116 sequences, 42 contigs were assembled using the SeqMan II program. In the absence of a reported *S. furcifera* genome sequences, the proteins derived from the yeast two-hybrid assay were identified by their similarity to available sequences in a BLAST search of the NCBI database. Totally 26 contigs were annotated, and 16 contigs were not able to annotate. Of those non-annotated, we did not know whether there was a protein present and whether it was unique to *S. furcifera*. Related contents appear in Table S3 and in lines 175-180.

o On line 178, you mention the 92.6% match to the N. lugens transcript, but the table lists a different match. Does this indicate that higher matches can be found to other species?

Response: BAP31 of *S. furcifera* has the highest similarity with the counterpart of *N. lugens*. We have corrected it in the Table S3. We also added this information in Fig. S2.

o On line 179, is there a specific domain of BAP31 that could be identified, or did you always pull-down the whole thing?

Response: The library screen identified the full-length sequence of BAP31 of *S. furcifera* interacted with P7-1 of SRBSDV. We didn't identify a specific domain of BAP31 that interacted with P7-1. We identified that the N-terminus of P7-1 interacted with the full-length sequence of BAP31 (684 bp). The related contents appear in Figure 2.

o Also, include the rationale for saying that the specific domains of DNAJB11 were interacting, is this because you can see specific domains in Y2H or did you actually do a split with domains to check yourself?

Response: The library screen found that the C-terminus of DnaJB11 of *S. furcifera* interacted with P7-1 of SRBSDV. We thus further used yeast two-hybrid assay to identify that P7-1 interacted with the C-terminal peptide-binding domain of DnaJB11, but not with other domains of DnaJB11. Related contents appear in Table S3, Figures 2A, 2B, and S3 and in lines 178-180 and 186-188.

- Line 194- It would probably be better to say "Low levels of binding by DNAJB11 antibodies was observed".

Response: We have revised this in lines 254-255 in revised version.

- Line 196- Expectedly, BAP31 antibodies did not recognize the specific inclusions in non-viruliferous or viruliferous insects. This line is confusing. Were we expecting inclusions in the non-viruliferous insects at all? Can you clarify this statement?

Response: We have revised this sentence as "Expectedly, only low specific staining in the cytoplasm was observed by BAP31 antibodies in nonviruliferous or viruliferous insects (Fig 5E)." in lines 257-259. We also changed the new Figure 5E.

- Line 303: "DNAJB11 and BAP31 have form an interesting interplay…" This should be either, 'DNAJB11 and BAP31 have formed an interesting interplay', or you could just delete the 'have'.

Response: We have revised this.

- Line 370- Define your kit, Also it should be ThermoFisher Scientific now that they have merged into one company.

Response: We have revised this in the full text.

- Line 384- I am just curious, how do you get exactly 400 second instars? Are you hatching the eggs on special media, is it coming out of rice, is this cited somewhere?

Response: We have revised it as “more than 400 second instars individuals” in line 446. The insects were collected after hatching on rice leaf sheath.

- Line 404- Figure numbers should be included here too.

Response: Line 404 in old manuscript has not the Figure number-related information.

Line 443- It usually that cells are ‘stained’ with dyes, not inoculated.

Response: We have revised this in the full text.

- Line 722 and Line 735- Are these alignments shown here with the top 5 hits? What was the reason these species were included in this alignment? Also, is it possible to move S. furcifera to a consistent placement in the alignment, always show on top or something, although the red is helpful.

Response: The sequence alignments were revised with the BAP31 and DnaJB11 amino acid sequences from *S. furcifera, N. lugens, L. striatellus, D. melanogaster* and *A. aegypti*. We have moved *S. furcifera* to a top placement in the two alignments. We think the comparison of BAP31 and DnaJB11 sequences among the three rice planthopper species and two model insects *D. melanogaster* and *A. aegypti* is better.

- Figure 6—Make sure your backgrounds are more consistently black, you have a lot of gray backgrounds indicating the contrast has been modified, please fix these panels, specifically Part A, column 2, Part B column 1-3.

Response: We agree with comment and followed. We have adjusted the backgrounds in Figure 3 in the revised version (Figure 6 in the old manuscript).

- Do you have brightfields for Figure 6? Or did you DAPI stain anything? It would be helpful to see the nucleus here to ensure that it is specifically cytoplasmic. My guess is that the void is nucleus, but I can’t tell without brightfield or without DAPI stain. Figure 7 is the same.

Response: We have added the brightfields in Figures 3 and 4 (Figures 6 and 7 in the old manuscript).

- Figure 8 is nice, but funny, as the nucleus is pretty huge in these cells, looks small here.

Response: We have used the new Figure 8.

- Figure S2 and S3—Again, define, are these the top hits and does this explain why they are not the same insects between the two proteins.

Response: We have chosen the same insect species, containing two top hits (*N. lugens* and *L. striatellus*) and two model insects (*D. melanogaster* and *A. aegypti*) in Figures S2 and S3.

- Table S3- This has the potential to be a truly fabulous table, as is, it is not useful. Please include the following columns- length of the protein-being sure to mark if you think you have full-length or partial- The percent match to the highest hit, the e-value. Asterisk those that you used in the study for confirmation.

Response: We have added length of the protein (full-length or partial), e-value and ORF integrality in Table S3.

- S4 table—remove the dashes in the column of the primers where no restriction enzymes are included in the primer. Mark the heading for restriction enzymes and add the footnote, "Where applicable".

Response: We have revised Table S4.

Reviewer #2: Minor suggestions:

1. Page 10, lines 285-286, the authors stated that "transmission capability of SRBSDV by S. furcifera is most efficient at the high temperature (35˚C)", I would suggest authors using "more" to replace "most".
2. Page 10, line 298, "play" is mistaken written as "paly".
3. Page 11, line 303, "form" should be replaced by "formed".

Response: We have checked and revised these minor mistakes in the revised version.

4. At 35 ˚C, SRBSDV acquisition rates by S. furcifera increases to >83%, and the transmission efficiency of one individual S. furcifera reached almost 89%. This is an important finding that will definitely help other groups to improve the transmission assay for this virus. I suggest this an important observation worth including in the abstract.

Response: We have described this result in the abstract in lines 34-35.

5. The prevalence of SRBSDV only restricted in the southern portion of the Yangtze River is not only caused by more efficient transmission capacity of S. furcifera at higher temperature, but also should consider the impact from rice subspecies and cultivars grown in these areas. The authors should also add this in the discussion or show evidence to support your statement.

Response: We have added other factors such as rice cultivars and cropping system in the Discussion section. The related contents appear in lines 311-314.

Reviewer #3: -L32. I am not sure if it is true that "hot environmental temperatures generally drive…". Perhaps this statement should be down toned.

Response: We have revised this sentence in the abstract in lines 27-28.

-L38. "… are activated…", by what?

Response: BAP31 and DnaJB11 are activated by viral infection. We have revised this in the abstract in line 33.

-L95-99. Several examples of mosquito-transmitted viruses are mentioned. But, what about insect-transmitted plant viruses? Add references if available. If not, mention that no information is available for plant virus transmission in relation to temperature.

Response: In the field, many insect-borne crop viral diseases are more suitable for maintenance and spread in tropical and sub-tropical climates, such as the brown planthopper *Nilaparvata lugens*-transmitted rice ragged stunt virus and rice grassy stunt virus, the white-backed planthopper *Sogatella furcifera*-transmitted southern rice black-streaked dwarf virus, and whitefly *Bemisia tabaci*-transmitted cassava mosaic virus. We have included this information in lines 85-89.

-L121. State here that SRBSDV is able to replicate in the insect vector.

Response: We have revised this in line 119.

-L124. … the epidemics of SRBSDV are restricted…

Response: We have revised this in line 122.

-L223. adaption > adaptation

Response: We have revised this in line 292.

-L303. have form > have formed

Response: We have deleted this.

-L330. papillomavirus > papillomaviruses

Response: We have deleted this.

-Table S1. Explain what I/II/III means. Explain how infection rates were calculate. Also, the infection rate results could be included as a figure, next to Figure S1.

Response: We have used the Expt I-III to replace I-III in Tables S1 and S2. "No. of positive insects with P7-1 in different tissues (n=30)" in the Table S1 is used to calculate infection rates. The infection rate results also have been included in Figure S1E.

-Fig. 1. I think that I/II/III/IV could be deleted as this labelling is redundant with 15/10/25/35. Also, both me and vm should be shown in all four photos, as I assume they show similar views.

-Fig. 3. "mg" could be deleted from the labelling. Arrows do not point to gold particles, fix this.

-Fig. 4. "vm" could be deleted from the labelling.

-Fig. 6. Roman numbers (I to VII) could be deleted from the labelling. They are redundant with the rest of information. Arrows do not point to gold particles, fix this.

Response: We agree with these comments and followed. We have carefully checked and revised the minor errors in the full text.
