# Supplementary material for: A plant reovirus hijacks endoplasmic reticulum-associated degradation machinery to promote efficient viral transmission by its planthopper vector under high temperature conditions
Source: PLoS Pathog. 2021 Mar 1;17(3):e1009347. doi: 10.1371/journal.ppat.1009347 (PMC7951979; doi:10.1371/journal.ppat.1009347)
Supplement: Attachment — Submitted filename: Response to reviewers.doc [file ppat.1009347.s012.doc]

**Point by point to Comments**

We would like to thank the editor and three expert reviewers for their insightful comments and suggestions on the manuscript, as these comments and suggestions led us to an improvement of the manuscript. Our revision reflects all suggestions and comments. Detailed responses to the editors and reviewers are given below.

Reviewer's Responses to Questions

**Part I - Summary**
Please use this section to discuss strengths/weaknesses of study, novelty/significance, general execution and scholarship.

Reviewer #1: The authors report that a plant virus SRBSDV is able to hijack the ERAD pathway in vectors by suppressing BAP31, which negatively regulates a heat shock protein (DnaJB11), and therefore ensure the proper assembly of tubules needed for viral transmission under high temperatures in vectors. They used appropriate methodologies to support their claims, such as Y2H, co-IP, as well as molecular and cellular imaging approaches. In addition, RNAi was also conducted to knock down DnaJB11 and BAP31 expression both in vitro and in vivo. The evidences are so strong that the causality of virus infection, high temperature tolerance, and DnaJB11 and BAP31 proteins are well demonstrated and clear. Although roles of BAP31 and DnaJB11 in virus transmission have been reported before, this research does deepen our understanding of temperature-mediated virus resistance. The manuscript is in general well written and figures are correct. All reviewers’ comments are responded very well. I believe that this work is extensive and systematic and is acceptable to PLOS Pathogens.

Response: Thank you for your positive envaluation of our work.

Reviewer #2: Yu et al. report that southern rice black streaked dwarf virus (SRBSDV) could hijack endoplasmic reticulum-associated degradation (ERAD) machinery for ensuring the proper assembly of abundant virus-induced tubules necessary for the efficient transmission of the virus by Sogatella furcifera in the manuscript entitled with "A plant reovirus hijacks endoplasmic reticulum associated degradation machinery to promote efficient viral transmission by its planthopper vectors under high temperature conditions". The manuscript is clearly written and contain very few spelling and grammatical errors. Some easily editable typos remain. The manuscript present a series of beautiful experiments.

However, the first section of results is titled “Infection efficiency of SRBSDV in S. furcifera increases with the elevated temperatures”. The first sentences of this section are a soup of ideas going from the viral nonstructural protein P7-1 and their role to the effect of temperature on SRBSDV infection in S. furcifera. This section doesn’t really discuss the infection efficiency but the accumulation of non-structural viral proteins. This part also describes the effect of temperature in the vector fitness. Please reorganize and/or streamline.

Response: We have reorganized the first section of results with the tittle “Effects of different temperatures on the development and viral infection of *S. furcifera*”. Vector-borne viruses establish their initial infection in the insect midgut, disseminate to the hemolymph and finally into the salivary glands, from where they are introduced into susceptible hosts. We investigated the effects of different temperatures on SRBSDV infection in the midguts or salivary glands of *S. furcifera* by immunofluorescence microscopy using virus-induced tubule and viroplasm as the markers. No. of insects positive for P7-1 and P9-1 of SRBSDV in different tissues under different temperatures at 6 days padp (n=30) has been calculated. The related contents appear in Figure 1A-C and Table S1.

No statistics are shown for the comparisons, therefore those cannot be assessed. Fig S1, legend describes letters for post-hocs, however images have *. Please correct. It is not clear what the * mean in regards to the comparisons, so the results and conclusions can’t be analyzed or interpreted. Further, for the effect of the temperature in insect fitness, no comparisons were performed between viruliferous and aviruliferous insects. So, sentences such as line 157 “However, under the high temperature (35˚C) for 6 or 7 days, the mortality rate of viruliferous S. furcifera was lower than that of nonviruliferous S. furcifera” are not supported by the data.

Response: We have carefully checked the full text and revised the description of statistics. We have compared the eclosion and mortality rates between viruliferous and nonviruliferous insects at 6 days in Figure S1E, F. Nonviruliferous or viruliferous fourth- to fifth-instar nymphs of *S. furcifera* were treated with temperatures at 15˚C, 20˚C, 25˚C, or 35˚C for different days, and the eclosion and mortality numbers were calculated daily. Each test contained three replicates and each replicate contained about 100 individuals of *S. furcifera*. Means (±SD) from three biological replicates are shown. The statistical significance is related to the 25˚C control. **P*<0.05. The related contents appear in Figure S1 and its legend, and in Lines 150-159 of Results section.

Fig S1 E is supposed to represent infection rate of different organs of the vector. I do not see how the graph reports that: what organs? How many insects? Which sex, stage, etc? All this section needs serious editing.

Response: We have deleted the Figure S1E in the original version. We added the new Figure 1C in the revised version to show “percentages of insects positive for P7-1 and P9-1 of SRBSDV in various tissues (midgut epithelium, midgut muscle, and salivary gland) under different temperatures (15, 20, 25 and 35˚C) at 6 days padp (n=30), as detected by immunofluorescence microscopy.” Based on our several previous published papers on SRBSDV-*S. furcifera* system, the female and male insects have similar infection or transmission efficiency by *S. furcifera*. The related contents appear in Figure 1A-C and Table S1, and in lines 161-176 of Results section, and in lines 491-502 of Materials and Methods section.

The authors then describe the identification of protein interacting with by Y2H. There is no information about the library that was screened. Please report what was used as RNA source. There is no information as to if the library was screened by mating or co-transformation. The authors do not mention testing for autoactivation of the system with either the prey or the bait, which is a serious problem of the Y2H system. This is particular necessary since the authors identified interactions with proteins containing membrane domains. One of the drawbacks of the Matchmaker system used is that it CANNOT detect interactions with membrane proteins. I appreciate the efforts of the authors performing the validations by Co-IP, however, the identified proteins are what could be considered “sticky” which might randomly interact with proteins. The co-localizations help support the argument for interaction.

Response: We have added the information about the library, method for the screening of library, and autoactivation test in Figure S4A. The related contents also appear in lines 434-452 of Materials and Methods section.

Meanwhile, the DUAL membrane system, a variant of the yeast two-hybrid assay to study membrane protein interactions, was also used to confirm the interactions among BAP31, DnaJB11 and SRBSDV P7-1 (Figure S4B). We also added the GFP as a negative control in Co-IP assay (Figure 2F). The co-localizations of interacting proteins *in vivo* or *in vitro* were shown in Figures 3 and 5.

Finally, the authors perform a series of experiments to confirm the potential role of the identified proteins in the formation of the virus-induced tubules. The work is interesting but remains a correlation. I am not certain that sentences such as (line 285) “it can be concluded that viral infection significantly upregulated DnaJB11 expression but inhibited BAP31 expression to benefit the assembly of SRBSDV P7-1 tubules for efficient viral transmission by S. furcifera” is supported by the data. The causation (mechanism by which the virus manipulate gene expression) was not identified. The other thing that I missed was the description of the feeding behavior of the insect at different temperatures, which of course can alter acquisition and inoculation.

Response: We have deleted this sentence in the revised version. We kept the sentence as the conclusion “These results suggested that a fine-tuned regulation of BAP31 and DnaJB11 expression is beneficial for the formation of P7-1 tubules, finally facilitating viral transmission by *S. furcifera*.” in lines 303-305.

Thank for your raising the important point. The Sf9 cells experiments in Figures 3 and 4 show “[Antagonistic regulation of](https://www.sciencedirect.com/science/article/pii/S0092867407009713) the assembly of SRBSDV P7-1 tubules by DnaJB11 and BAP31”. In the Discussion section, we carefully analyze the causation of the different functions of two proteins BAP31 and DnaJB11 in the process of tubule formation: “SRBSDV P7-1 is initially retained in the ER, and then secreted into the cytosol to assemble the tubules. BAP31 could act as an ER retention “receptor” for P7-1 by forming a complex with P7-1. DnaJB11 competes with BAP31 for binding P7-1, and thus the overexpression of DnaJB11 leads to the delivery of P7-1 from BAP31-P7-1 complex in the ER to DnaJB11-P71 complex. This process finally regulates the export of P7-1 from the ER and ensures the proper assembly of tubules. We thus deduce that BAP31 as a sorting factor may just transiently retain the newly synthesized P7-1 in the ER for further folding and assembly, and then deliver it to DnaJB11-containing chaperoning complex that determine the fate of P7-1. SRBSDV infection inhibits BAP31 expression but upregulates DnaJB11 expression, while BAP31 negatively regulates the expression of DnaJB11 through their direct interaction. Thus, the down-regulation of BAP31 expression during viral infection in turn promotes DnaJB11 expression and tubule assembly, finally facilitating viral transmission by *S. furcifera*. Taken together, we show that a fine virus-mediated regulation of protein quality control has evolved for activation of DnaJB11 for ensuring the proper assembly of virus-induced tubules to support viral propagation and transmission by insect vectors.” The related contents appear in lines 348-368 of Discussion section.

It is possible that different temperatures may affect the feeding behavior of the insects and viral acquisition. Thus, in the revised version, we deleted the related descriptions for the effect of temperatures on viral acquisition. Vector-borne viruses establish their initial infection in the insect midgut, disseminate to the hemolymph and finally into the salivary glands, from where they are introduced into susceptible hosts. We investigated the effects of different temperatures on SRBSDV infection in the midguts or salivary glands of *S. furcifera* by immunofluorescence microscopy using virus-induced tubule and viroplasm as the markers. No. of insects positive for P7-1 and P9-1 of SRBSDV in different tissues under different temperatures at 6 days padp (n=30) has been calculated. The related contents appear in Figure 1A-C and Table S1.

Reviewer #3: This is a good manuscript contributing to our understanding the transmission of plant pathogens by insect vectors. Specifically, the involvement of ER and ER-related proteins and mechanisms (like ERAD) in the transmission. The involvement of ER mechanisms in the transmission is not new and it has been shown for other systems although those two proteins are new.

Response: Thank you for your positive envaluation of our work.

**Part II – Major Issues: Key Experiments Required for Acceptance**
Please use this section to detail the key new experiments or modifications of existing experiments that should be absolutely required to validate study conclusions.

Generally, there should be no more than 3 such required experiments or major modifications for a "Major Revision" recommendation. If more than 3 experiments are necessary to validate the study conclusions, then you are encouraged to recommend "Reject".

Reviewer #1: None.

Reviewer #2: Statistics of the experiments need to be better described and results of the analyses reported.

Response: We have checked and revised the description of statistics in the full manuscript.

The Y2H system needs to have the proper controls. Test for autoactivation and I recommend testing bait and prey with unrelated proteins (can be the ones that come as controls with the kit). Same for the co-IP. For the silencing, I did not see the expression of the untreated insects, sometimes there is an effect with the GFP control.

Response: We have added the test for autoactivation during library screening in Figure S4A. We used several different unrelated insect proteins such as DnaJA1, DnaJA2, Derlin-1, Derlin-2, Hsp68 and BiP, to test the specificity of yeast two-hybrid assay in Figure S4C. Meanwhile, the DUAL membrane system, a variant of the yeast two-hybrid assay to study membrane protein interactions, was also used to confirm the interactions among BAP31, DnaJB11 and SRBSDV P7-1 (Figure S4B). We also added the GFP as a negative control in Co-IP assay (Figure 2F). The co-localizations of interacting proteins *in vivo* or *in vitro* were shown in Figures 3 and 5. For the silencing experiment in Figure 6, we have added the untreated insects as alternative controls.

Reviewer #3: The current manuscript suggests an interesting hypothesis by which a plant virus, southern rice black-streaked dwarf virus, SRBSDV, which is transmitted by the planthopper Sogatella furcifera in rice uses ER machinery to promote its own transmission under high temperatures. With 35 degrees the acquisition and transmission of the virus is significantly increased. This is achieved by the overexpression of DnaJB11 and BAP31 genes upon virus acquisition by the insect. DnaJB11 facilitates the formation of viral tubules with the P7-1 nonstructural protein of the virus which in turn facilitates transmission. The relationships between the two candidate proteins studied here are antagonistic, while fine tuned interactions ensure these functions in virus transmission. BAP31 negatively regulates the expression of DnaJB11 and under high temperatures BAP31 is negatively regulated while DnaJB11 is upregulated leading to higher transmission.
The authors have performed a wealth of experiments, and I must say mostly in vivo experiments to demonstrate the relationships between P7-1, DnaJB11 and BAP31 and the formation of viral structures for transmission. Those experiments included expression at the gene and protein levels, in vitro interactions using pull down assays and Y2H system and interaction at the cell culture levels using SF9 cells. In the SF9 system, knockdown assays were used to demonstrate these relationships.
The quality of the experiments is convincing and no doubt that both proteins influence the virus dynamics in the insect as demonstrated in the immunofluorescence, interaction and knockdown experiments in both dissected organs and cell cultures.

Response: Thank you for your positive envaluation of our work.

The main drawback which I think is a must for accepting this manuscript is the ability of the authors to demonstrate the role of these interactions in the virus transmission by the insect, meaning transmission experiments following introducing interference like knockdown or specific antibodies. This is the ultimate result which should demonstrate that upon knockdown of one or both of these proteins in the insect, the transmission is affected. I am not sure whether such methods are developed for this insect-virus system, but as judged from other systems, I guess it should not be a problem. The demonstrated interaction are all in vivo interactions which might involve other proteins that influence the virus persistence in the insect, suggesting that the two identified proteins have a secondary role in the transmission. It is known from other pathogen-vector systems that the ERAD machinery influences several steps in the transmission of pathogens, and with this machinery, many proteins are involved. It cannot be ruled out that some of the observed interactions shown in this manuscript involve other factors that have not been identified here, which have a more important role than the ones identified here. As mentioned above, several ER-related proteins function in the ERAD machinery. Although only the two proteins studied here were found to interact in the initial screen, the authors could test other ER and membrane associated proteins which could function exactly as these two proteins. Since the system has been established and these confirmation methods have been used, other candidates could easily be screened.

Response: Thanks for your valuable suggestions. The ERAD system contains several ER-related proteins, such as DnaJB11, BAP31, DnaJA1, DnaJA2, Derlin-1, Derlin-2, and ER Hsp70 chaperone BiP. DnaJB11 generally binds to the membrane proteins and delivers them to BiP for ATP-dependent chaperoning; however, DnaJB11 also can binds directly to unfolded protein clients in the ER without delivering to Bip. BAP31 binds to newly synthesized membrane proteins to ER membrane through the direct interaction of BAP31 with Derlin-1 or translocon-associated components. Derlin-1 is also among the hits in our yeast two hybrid screen, whereas other ERAD components are not. We have confirmed that DnaJA1, DnaJA2, Derlin-1, Derlin-2, Hsp68, and BiP of *S. furcifera* did not directly interact with SRBSDV P7-1 (Figure S4C). How the ERAD machinery manages to handle a substrate as big as the P7-1 tubules of SRBSDV remains an intriguing question that may be answered by more detailed analysis of BAP31, DnaJB11 and ERAD-associated Hsp chaperones. The related contents appear in Figure S4C, in lines 219-223 of Results section, and in lines 370-381 of Discussion section.

The mechanisms by which the interactions between the two candidates are happening, how their antagonistic relationships are occurring are yet not understood, and the manuscript lacks this significant addition to understanding the transmission. One hypothesis might be other HSPs? Why other such proteins were not tested as it is known from other works?

Response: Thank for your raising the important point. The Sf9 cells experiments in Figures 3 and 4 show “[Antagonistic regulation of](https://www.sciencedirect.com/science/article/pii/S0092867407009713) the assembly of SRBSDV P7-1 tubules by DnaJB11 and BAP31”. In the Discussion section, we carefully analyze the antagonistic relationship of two proteins BAP31 and DnaJB11 in the process of tubule formation: “SRBSDV P7-1 is initially retained in the ER, and then secreted into the cytosol to assemble the tubules. BAP31 could act as an ER retention “receptor” for P7-1 by forming a complex with P7-1. DnaJB11 competes with BAP31 for binding P7-1, and thus the overexpression of DnaJB11 leads to the delivery of P7-1 from BAP31-P7-1 complex in the ER to DnaJB11-P71 complex. This process finally regulates the export of P7-1 from the ER and ensures the proper assembly of tubules. We thus deduce that BAP31 as a sorting factor may just transiently retain the newly synthesized P7-1 in the ER for further folding and assembly, and then deliver it to DnaJB11-containing chaperoning complex that determine the fate of P7-1. SRBSDV infection inhibits BAP31 expression but upregulates DnaJB11 expression, while BAP31 negatively regulates the expression of DnaJB11 through their direct interaction. Thus, the down-regulation of BAP31 expression during viral infection in turn promotes DnaJB11 expression and tubule assembly, finally facilitating viral transmission by *S. furcifera*. Taken together, we show that a fine virus-mediated regulation of protein quality control has evolved for activation of DnaJB11 for ensuring the proper assembly of virus-induced tubules to support viral propagation and transmission by insect vectors.” The related contents appear in lines 348-368 of Discussion section.

However, the mechanisms by which the antagonistic interaction relationship between BAP31 and DnaJB11 of *S. furcifera* occurs are yet not understood. Continued studies may confirm whether such antagonistic interaction of BAP31 and DnaJB11 is a common phenomenon in nature. We believe that the newly identified protein quality control pathway via regulation of BAP31 and DnaJB11 expression would play more important roles in ensuring the proper protein functions under different abiotic and biotic stress. The related contents appear in lines 381-387 of Discussion section.

Throughout the experiments controls were not included and those would be must. For example Figure 5D in which a negative control is not shown for this TEM experiment. Several other relevant controls with the immunocolocalization experiments were not included.

Response: We have checked and added the necessary controls in the revised version. For example, the nonviruliferous control of the TEM experiment in Figure 5D; The negative control of the TEM experiment in Figure 3J; The nonviruliferous control of the immunofluorescence microscopic experiment in Figure 1B.

We have added the test for autoactivation during library screening in Figure S4A. We used several different unrelated insect proteins such as DnaJA1, DnaJA2, Derlin-1, Derlin-2, Hsp68, and BiP, to test the specificity of yeast two-hybrid assay in Figure S4C. We also added the GFP as a negative control in Co-IP assay (Figure 2F). For the silencing experiment in Figure 6, we have added the untreated insects as alternative controls.

**Part III – Minor Issues: Editorial and Data Presentation Modifications**
Please use this section for editorial suggestions as well as relatively minor modifications of existing data that would enhance clarity.

Reviewer #1: Why do the authors use the fall armyworm cell lines when they have already developed the cell cultures of Sogatella furcifera (Jia et al., 2012. Journal of Virology, 86, 5800 - 5807).

Response: The cell cultures of *S. furcifera* are not suitable for transient expression of proteins, though they can be inoculated with viruses. The Sf9 cells are suitable for exogenous protein expression by the baculovirus expression vector system.

Line 313, SRBSDD should be SRBSDV.
Line 319, I prefer “it appears that SRBSDV infection improves the tolerance of S. furcifera to high temperature (36˚C)”.

Response: We have revised.

The authors have provided sufficient evidence that BAP31 negatively interacts with DanJB11. How do these proteins bind to each other? Would it be possible for authors to predict potential protein binding sites via bioinformatics analysis?

Response: We have identified that BAP31 interacted with the C-terminal peptide-binding domain of DnaJB11 by yeast two hybrid in Figure 2B. We would like to further determine the potential protein binding sites via bioinformatics analysis and other experiments.

Reviewer #2: Rearrange first section of results, complete materials and methods.

Response: Thanks for your valuable suggestions and we have followed.

Reviewer #3: None
