# Supplementary material for: A plant reovirus hijacks endoplasmic reticulum-associated degradation machinery to promote efficient viral transmission by its planthopper vector under high temperature conditions
Source: PLoS Pathog. 2021 Mar 1;17(3):e1009347. doi: 10.1371/journal.ppat.1009347 (PMC7951979; doi:10.1371/journal.ppat.1009347)
Supplement: Attachment — Submitted filename: Response to reviewers-1.doc [file ppat.1009347.s013.doc]

**Point by point to Comments**

We would like to thank the editor and three expert reviewers for their insightful comments and suggestions on the manuscript, as these comments and suggestions led us to an improvement of the manuscript. Our revision reflects all suggestions and comments. Detailed responses to the editors and reviewers are given below.

Reviewer's Responses to Questions

In the manuscript, you have emphasized that insect proteins have evolved to ensure the proper assembly of virus-induced tubules to support viral propagation and transmission by insect vectors (e.g. L44-47 in the abstract; L55-56 in the author summary; L365-368 in the discussion). However, I don’t think this is the case. In general, the insect doesn’t evolve to ensure virus infection. In addition, in several places, generic conclusion was made based on studies of one virus (SRBSDV), e.g. L47-48 in the abstract; L58-60 in the author summary and L385-387, L400-410 in the discussion. The authors need to re-phrase these sentences.

Response: Thank you for your positive envaluation of our work. We have re-phrased the sentences of L45-48 in the abstract, L59-63 in the author summary, L372, L393-394 and L408-409 in the discussion.
